# Pica and Amylophagy Are Common among Malagasy Men, Women and Children

**DOI:** 10.1371/journal.pone.0047129

**Published:** 2012-10-17

**Authors:** Christopher D. Golden, B. J. Rodolph Rasolofoniaina, Rakoto Benjamin, Sera L. Young

**Affiliations:** 1 Harvard University Center for the Environment, Cambridge, Massachusetts, United States of America; 2 Madagascar Health and Environmental Research (MAHERY), Maroantsetra, Madagascar; 3 Départemente d'Agriculture, École Supérieure des Sciences Agronomiques, Université d'Antananarivo, Antananarivo, Madagascar; 4 Division of Nutritional Sciences, Cornell University, Ithaca, New York, United States of America; Indiana University, United States of America

## Abstract

Pica, the craving and purposive consumption of non-food substances, is of public health concern for its potential deleterious and salubrious health consequences. However, neither its prevalence nor demographic correlates have been well characterized. Therefore, we conducted the first population-based study of pica and amylophagy in Madagascar. From February to December 2009, we surveyed pica and amylophagy behaviors in a random sample of 760 individuals >5 years in 167 households among two ethnic groups in 16 villages in the Makira Protected Area of Madagascar. Of the 760 individuals interviewed, 62.5% were children (5–11 years), 5.4% were adolescents (12–16 years), and 35.1% were adults (≥17 years). Thirteen non-food items were reported being consumed. Across the entire population in the prior year, the prevalence of geophagy was 53.4%, of amylophagy, 85.2%, and of other pica substances (e.g. charcoal, chalk) was 19.0%. The prevalence of these behaviors was not higher during pregnancy. These findings differ from previous studies in terms of the higher overall prevalence of these behaviors, the high prevalence among men, and the absence of any peak in behaviors during pregnancy. However, there are two categories of substances that elevate our estimates but fall outside the strict definition of pica as a craving: 1) substances consumed for self-medication and 2) substances viewed as food, such as all amylophagic substances in this case. Our results suggest that population-based studies of pica should include males of all ages. Further, the prevalence of the behavior underscores the importance of understanding the etiology and health consequences of these ingestive behaviors (Abstract S1).

## Introduction

Pica is the craving and purposive consumption of substances not culturally defined as food [1]. While pica can imply the consumption of a range of substances, geophagy, the consumption of earth, and amylophagy, the consumption of raw starches, are among the most common types of pica [2]–[4]. Pica is of public health interest because of its potentially positive and negative health consequences [5]–[7]. In terms of benefits, it may protect against harmful pathogens and toxins; quell nausea, vomiting and diarrhea; or contribute beneficial nutrients. Pica may also be harmful, by reducing the bioavailability of beneficial nutrients, introducing toxic substances, or by acting as a vector for geohelminth infection. Further, it is of public health interest because it is highly prevalent among the most biologically vulnerable populations: pregnant women and children [1].

One reason that pica remains poorly understood is that neither its prevalence nor social and biological correlates have been well characterized. Pica is frequently either overlooked by researchers, concealed by consumers, or both [7]. While the bulk of the reports of pica have been anecdotal, the prevalence of pica in population-based studies has begun to be reported in the last few decades, mostly among pregnant women [8]–[14] and children [15]–[19]. These more recent data are a welcome complement to the many ethnographic studies that provided general information indicating that pica was common around the world [20]–[23].

Our purpose is two-fold. The first is to present the first reported evidence of the prevalence of pica and amylophagy in Madagascar. We separate amylophagy as a distinct category because it is culturally viewed as food in Madagascar. Our second purpose is to characterize the demographic correlates of pica and amylophagy in Madagascar. These data will permit elucidation of potential etiologies and health consequences of this enigmatic behavior. Indeed, to date, the only evidence of human geophagy in Madagascar is from a 17^th^ century tome written by the then governor of Madagascar, a French national, Étienne de Flacourt [24]. In it, he recounted how three types of earth were consumed in Madagascar:

“Il y a de diverses sortes d'excellent bol & de la vray terre sigillée, aussi bonne que celle de l'Isle de Lemnos, & le bol est aussi fin que celuy d'Armenie, le bol rouge se nomme *Tanemene*, & la terre sigillée, *Tanelisse*. Il y a une terre blanche comme de la craye, que est tres-excellente à degraisser & savonner le linge, elle est aussi bone que le savon. Elle est grasse & argilleuse, & semblable à la terre te Malthe, que l'on vend en France, à qui on attribuë la faculté de tuer & chasser les serpens, & resister à leurs venins, elle se nomme *Tanefoutchi*.”

The clays were described as being similar to the highly prized and very expensive medicinal earths consumed at that time in Europe and the Middle East, including Lemnian and Armenian “stamped” clays, known as terra sigillata [25]. The three types of earth consumed were *tanemene* (described by de Flacourt as a red clay), *tanelisse* (described as a white clay), and *tanefoutchi* (described as a white, greasy clay useful for laundering and similar to a clay sold in France to keep away snakes and treat their bites). However, neither he nor subsequent scholars of Madagascar have offered any suggestion of the prevalence of their consumption. Indeed, the absence of any description of human geophagy in Madagascar is striking, given that geophagy has been observed among nine species of non-human primates there [5].

## Methods

### Study Site

This study was conducted in 16 villages throughout the Makira Protected Area, a forest in northeastern Madagascar. This forest covers 371,217 hectares of lowland and mid-altitude rainforest, and represents the largest remaining tract of contiguous forest in Madagascar [26]. It is one of the most biologically diverse ecosystems in Madagascar and is home to two of the 18 major ethno-linguistic groups in Madagascar, the Betsimisaraka in the east and south, and the Tsimihety in the north and west [26]. Both the Betsimisaraka and Tsimihety in this region are almost exclusively agriculturalists. The Tsimihety tend to inhabit the more interior, harder to reach regions within the Makira Protected Area. Although in other areas of Madagascar these two ethno-linguistic groups are more distinct, where they have come to intermingle, such as in the Makira Protected Area, ethnic differences are minimal.

### Data collection

To ensure that the surveys would query relevant pica items, we first conducted six focus group discussions to generate a list of commonly consumed “non-food” items. We also added raw starches that people did not list, knowing that other cultures view amylophagy as a type of pica. Focus group discussions were conducted with three different age groups (children 5–11 years, adolescents 12–16 years and adults older than 16 years) in two different villages among both Betsimisaraka and Tsimihety. Each focus group consisted of six people, three men and three women. We then piloted the survey in 10 households in one village and revised the survey accordingly.

From February to December, 2009, a local female nurse and local male physician then surveyed 760 males and females from 16 villages in the Makira Protected Area. Households were sampled from each village using systematic random sampling from a census list generated by the research team. The research team was familiar with this region through work with a larger environmental and health monitoring research project in the area [27]. The survey was administered to every member of the household who was older than five years of age. Children were surveyed in exactly the same way as adults, i.e. the purpose of our research was discussed and then they were asked about their pica behaviors across a list of predetermined substances. Surveys lasted 10–30 minutes and were conducted in respondents' homes in Betsimisaraka or Tsimihety, the local languages.

The survey covered numerous topics, including types of pica and amylophagic substances consumed in the past year, the geographical location of their source, methods of preparation, price (if purchased), motivation for consumption, and the amount and frequency of consumption over the past year. For women, pregnancy status was verbally ascertained. When asking about the motivation for consumption, respondents were requested to first list the primary hedonic reason for consumption (i.e. taste, texture, smell, temperature, color, etc.) and then to offer any additional information. If a participant explained that the substance was used for medicinal purposes this was also noted. “Medicine”, *aody*, was used broadly to mean both a remedy used to cure an ailment as well as a spiritual substance used for protection. Open-ended questions permitted participants to report other substances that were not specifically inquired about or about periods in their life when they consumed these items but now no longer do so (i.e. during childhood, pregnancy, etc.).

The quantity consumed was inquired about after determining the frequency of consumption. Users were asked if the substance they ate was equivalent to a spoonful (approximately 15 mL), a *tasse* (an espresso-sized cup, standard in Malagasy households, approximately 100 mL), a handful (approximately 300 mL), or a cup (1 L plastic cup common in Malagasy households in this area).

### Variable creation and data analysis

We organized consumption into categories of “geophagy,” “amylophagy,” and “other non-foods,” including used coffee grounds, charcoal, pulverized rice chaff, rock salt, chalk and ash. “Other non-foods” included all pica substances that was not “geophagy.” The amylophagic items were not considered to be non-foods by consumers, so are assessed separately from those that fit the definition of pica. They remain included in our analyses because amylophagy has been considered to be pica in a number of other settings, e.g. the United States [28]–[30], Ghana [31], and Tanzania [1].

All surveys were entered into a Microsoft Access database and systematically spot-checked by two research personnel to assure correct input. Statistical analyses were conducted in STATA version 11 (College Station, TX). Chi-square tests were used to test for significant differences between consumption behaviors by group.

### Pica substance sampling

One author (CG) accompanied pica consumers to collect one sample each of select geophagic substances (*tany manara*, *vato malemy* and sand) following the protocol outlined by Young et al. [7]. Select physical analyses were then performed and analyzed in the soil science laboratory at the Department of Agriculture at the University of Antananarivo in Madagascar to determine clay content (by RB). Samples were mixed thoroughly and then dried at room temperature. After disaggregation with a porcelain mortar and wooden pestle, particles smaller than 2 mm were sieved and kept for physical analysis to measure particle size.

### Ethics Statement

**Figure 1:**
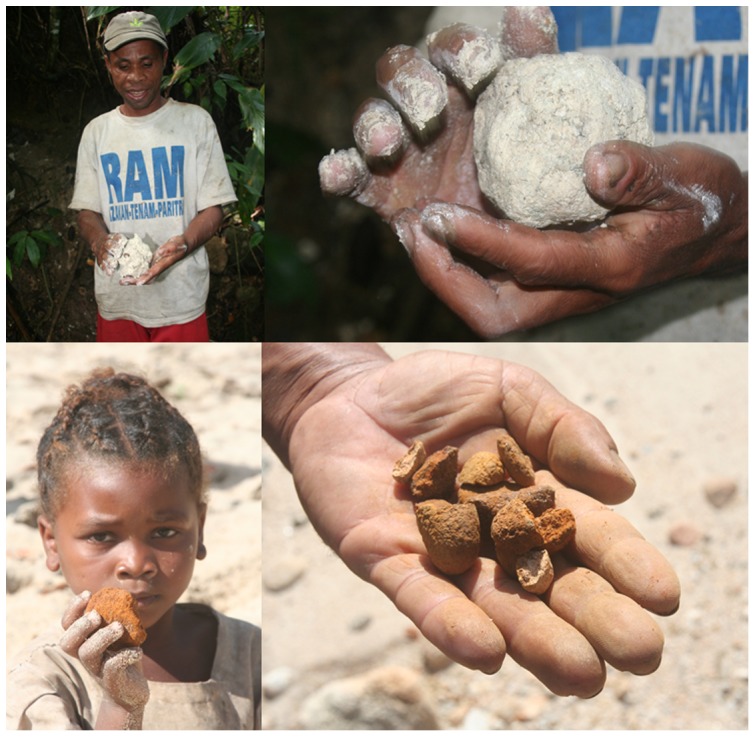
Geophagic substances. The two most commonly consumed geophagic substances are *tany manara* and *vato malemy*. A local healer prepares a bolus of *tany manara*, a white clay-like substance that has high content of calcium carbonate (top). A child holding *vato malemy*, reddish clods of earth that form along riverbeds (bottom).

This study was approved by the University of California, Berkeley's Committee on the Protection of Human Subjects (CPHS#2007-2-3), the Ministry of Water and Forests in Madagascar (#135/09/MEFT/SG/DGEF/DSAP/SLRSE) and the Maroantsetra District Hospital's Medical Inspector. We also obtained approval from the listed boards to receive oral informed consent from all study participants because there are illiterate members of the population for whom reading consent documents and signing their name would be impossible. For the two subjects of the photographs in Figure 1, we obtained written informed consent to publish them, as outlined in the PLOS consent form.

## Results

### Population characteristics

Of the 760 individuals interviewed, 62.5% were children (5–11 years old), 5.4% were adolescents (12–16 years old), 32.1% were adults (≥16 years), and 3.0% of the total population were adult females who were either pregnant or lactating. 48.6% were members of the Betsimisaraka ethnolinguistic group, and 51.5% were Tsimihety.

### Pica and amylophagic substances

Thirteen substances were reported being consumed (Table 1). The geophagic substances were *tany manara*, sand, and *vato malemy*. *Tany manara* is a white, calcium carbonate-based sub-soil typically found 1–2 meters below ground (Fig. 1). *Tany manara* resembles the description of *tanefoutchi* provided by de Flacourt [24]; tanefoutchi (modern spelling *tany fotsy*) is a regional dialect of Malagasy but both literally mean “white earth.” *Vato malemy* is river sediment that forms into small reddish-colored clods in riverbeds (Fig. 1). Sand was the direct local translation of what consumers described as the material that fell off visitors' feet in their homes. Select physical characteristics were determined for *tany manara*, *vato malemy* and sand. In terms of particle size, no geophagic substances were high in clay content (2.8%, 4.1% and 20.5% respectively). Of note, the item described as sand actually had the highest clay content.

**Table 1:** Prevalence of amylophagy, geophagy, and other non-food consumption during the prior one year among 760 randomly chosen Malagasy individuals.

Pica items	Population prevalence
**Geophagy**	53.4%
*Tany manara* (white sub-soil)	49.5%
Sand	4.7%
*Vato malemy* (red river sediment)	3.7%
**Amylophagy**	85.2%
Raw cassava	84.1%
Raw sweet potato	43.3%
Uncooked rice	20.8%
*Ambaradedin-ambazaha* (wild root)	9.6%
**Other pica**	19.0%
Rock salt	8.0%
Coffee grounds	7.5%
Charcoal	6.1%
Rice chaff	5.9%
Chalk	4.7%
Ash	2.0%

The amylophagic substances consumed were raw cassava, raw sweet potato, uncooked rice and *ambaradedin-ambazaha* (Table 1). A*mbaradedin-ambazaha*, a species from the genera *Hedychium*, is a type of root which is always consumed raw. Cassava, sweet potato and unfortified rice are grown locally and typically eaten cooked. Cassava, sweet potatoes and *ambaradedin-ambazaha* were typically washed and peeled before eating them raw.

There were also a number of non-food substances consumed that were not soils or sediments: rock salt, used coffee grounds, charcoal, pulverized rice chaff (i.e. the husk or scaly casing), chalk and ash (Table 1). Rock salt is locally produced in Madagascar and is sold unpackaged. Coffee grounds are typically consumed after preparing coffee from locally-produced beans. The chalk consumed locally does not have a brand name but is blackboard chalk typically from the local schools. Ash is collected from cooking fires after they have cooled; wood fires are made with a variety of locally collected wood. Pulverized rice chaff refers to the husks that which is separated from the rice grain after shelling rice with a mortar and pestle.

A variety of methods were used to prepare pica substances prior to consumption. *Tany manara* underwent the most complicated preparation process. It is typically washed and then molded into a sphere, which is then dried in the sun. Once it is dry, pieces are chopped (according to the dose suggested by a healer) and either consumed solid or mixed into water or coconut water. *Tany manara* was the only pica substance stored for consumption. It was often stored in woven baskets, plastic bags or folded into laundered shirts. Sand (left by people's feet) was typically taken off of household mats and then sifted so that only the smallest particles were consumed. Consumers described the “sand” brought in by visitors' feet as very fine topsoil particles and mentioned how they enjoyed consuming it. *Vato malemy* tends to be thought of as a “clean” substance since it is found by riverbeds, but some consumers washed it in the river before consuming it. Ash was either consumed as it was collected, or sifted and mixed with water and drunk. Chalk was often finely chopped or shaved into small pieces before eating. Charcoal was either broken into very fine pieces before consumption or was crushed and mixed with water, then drunk. Rice chaff was often further pulverized with a mortar and pestle before consumption.

All substances were common locally and available within close proximity to villages either from agricultural fields (cassava, sweet potato, rice and coffee), in river beds (*vato malemy*), in the forest (*ambaradedin-ambazaha*), or in households or schools (rock salt, chalk, ash, charcoal, sand and rice chaff), with one exception: *tany manara*. *Tany manara* was often found in the forest far from villages and required a knowledgeable healer to procure. Healers are local ethnomedical specialists that have immense knowledge of both botanical and other resources used for physical and spiritual health.

Only three pica substances were ever purchased (all prices that follow are in US Dollars). Chalk was either bought or stolen (more frequently the latter), and ranged in cost from $0.01–0.05 per piece. Rock salt was always purchased and cost $0.05 for a 150 g package. *Tany manara* was purchased from healers for $0.03–0.25 per bolus (approximately a handful).

### Patterns of consumption

The prevalence of geophagy across the entire population in the prior year was 53.4%, amylophagy was 85.2%, and other pica (not including geophagy or amylophagy) was 17.0%. Comparing the Betsimisaraka to the Tsimihety ethnolinguistic groups, the prevalence of geophagy (63.1% vs. 44.2%), amylophagy (90.0% vs. 80.8%) and other non-foods (25.2% vs. 12.8%) was significantly higher in Betsimisaraka people (χ2 p<0.0001 for all comparisons).

**Figure 2:**
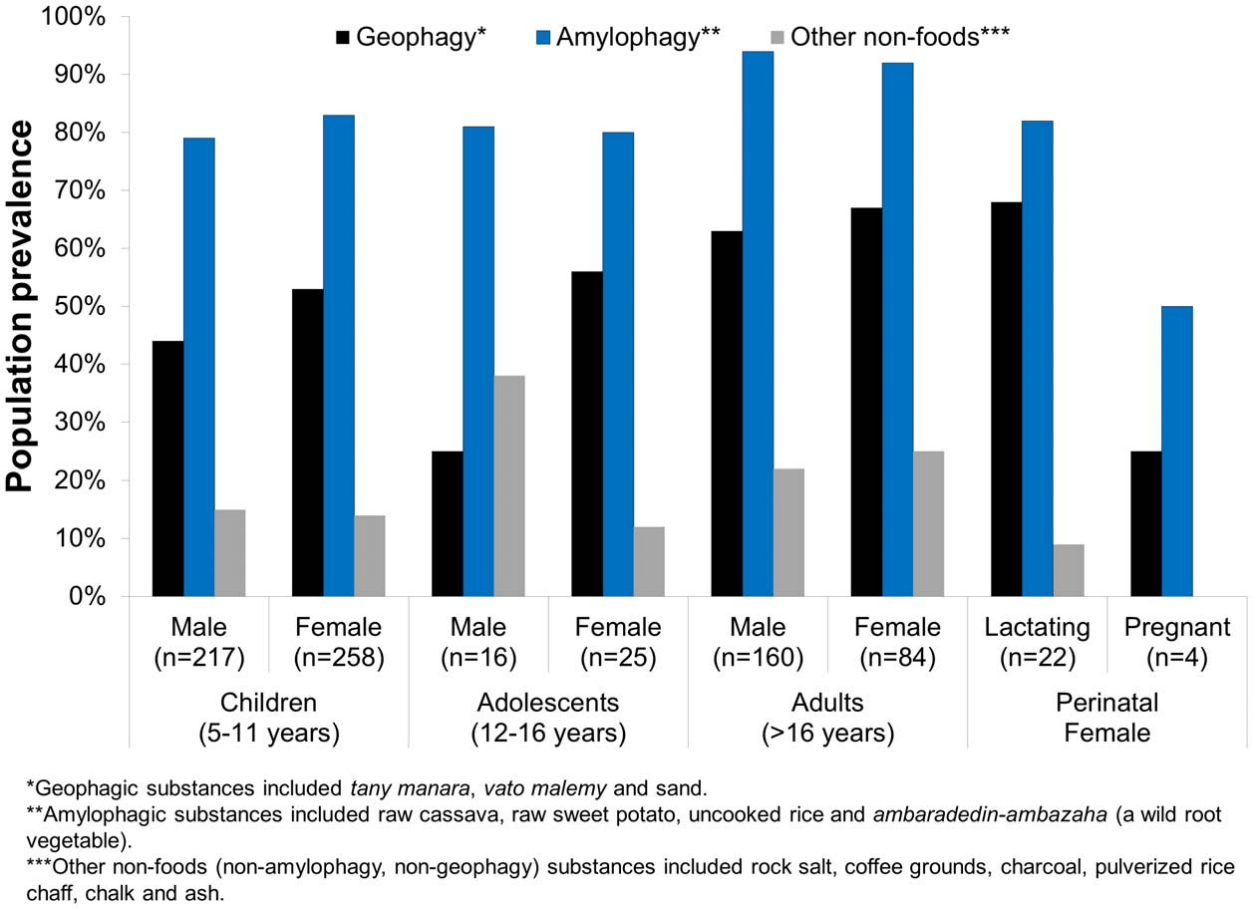
Prevalence of pica and amylophagy behavior by sex, age and pregnancy status among 760 individuals in rural, northeastern Madagascar. There were no significant differences of pica behavior by sex within age categories but adults practiced all pica behaviors significantly more than children or adolescents. Across all age and sex categories, amylophagy was practiced more widely than geophagy.

Neither the prevalence of geophagy nor of amylophagy was significantly different between sexes among children, adolescents or adults (only non-lactating adolescent and adult women included for this analysis, Fig. 2). The consumption of other non-foods (non-geophagic pica) was, however, significantly higher among male adolescents than among female adolescents (χ2 p = 0.041) but not different by sex among children or adults.

Adults practiced all forms of pica and amylophagy significantly more than children (Fig. 2; χ2: geophagy (p<0.0001), amylophagy (p<0.0001) and other non-foods (p = 0.009)) and adolescents (Fig. 2; χ2: geophagy (p = 0.015), amylophagy (p = 0.006) but not other non-foods (p = 0.931)). Interestingly, the prevalence of these consumption behaviors did not increase during pregnancy or lactation (Fig. 2; χ2: pica all categories (p = 0.307), geophagy (p = 0.225), amylophagy (p = 0.727)). When asking non-pregnant women about their pica behaviors during pregnancy, less than 1% of women reported consuming pica substances during pregnancy that they did not eat after delivery.

The quantity of substance consumed within a 24 hour period ranged from a fraction of a teaspoon (15 mL) to more than 1 liter (Table 2). Generally, the amylophagic substances were consumed in the largest quantities, whereas the geophagic substances were consumed in the smallest quantities (Table 2). In terms of frequency, substances were consumed from only once per month to as frequently as twice per week by those who ate them. Raw sweet potato and a local tuber (*ambaradedin-ambazaha*) were consumed with the least frequency, while sand and charcoal were consumed with the most frequency (Table 2), by those who ate them.

**Table 2:** Frequency and quantity of ingestive pica behaviors.

	Frequency/wk Mean (standard error)	Quantity consumed in 24 h Mode (range) in mL
**Geophagy**		
Sand	2.12 (1.65)	15 (15–150)
*Vato malemy* (red sediment)	0.79 (0.71)	15 (15–300)
*Tany manara* (white soil)	0.78 (0.84)	15 (15–50)
**Amylophagy**		
Uncooked rice	1.86 (1.05)	15 (15–300)
Raw cassava	1.46 (0.73)	300 (150–1000)
Raw sweet potato	0.50 (0.21)	300 (150–1000)
*Ambaradedin-ambazaha* (wild root)	0.23 (0.13)	100 (50–1200)
**Other pica**		
Charcoal	2.04 (1.22)	30 (15–600)
Chalk	1.06 (0.61)	15 (15–150)
Rock salt	1.02 (0.95)	4 (4–30)
Rice chaff	0.96 (0.62)	15 (15–300)
Coffee grounds	0.86 (0.50)	15 (15–300)
Ash	0.57 (0.08)	15 (15–300)

Although not quantified specifically in this study, our open-ended questions allowed for an understanding of the duration of consumption of certain items. Individuals would typically consume charcoal only when ill and those individuals consuming sand would only do so during very short periods during the year, unlike the other substances consumed frequently which often were lasting behaviors.

**Figure 3:**
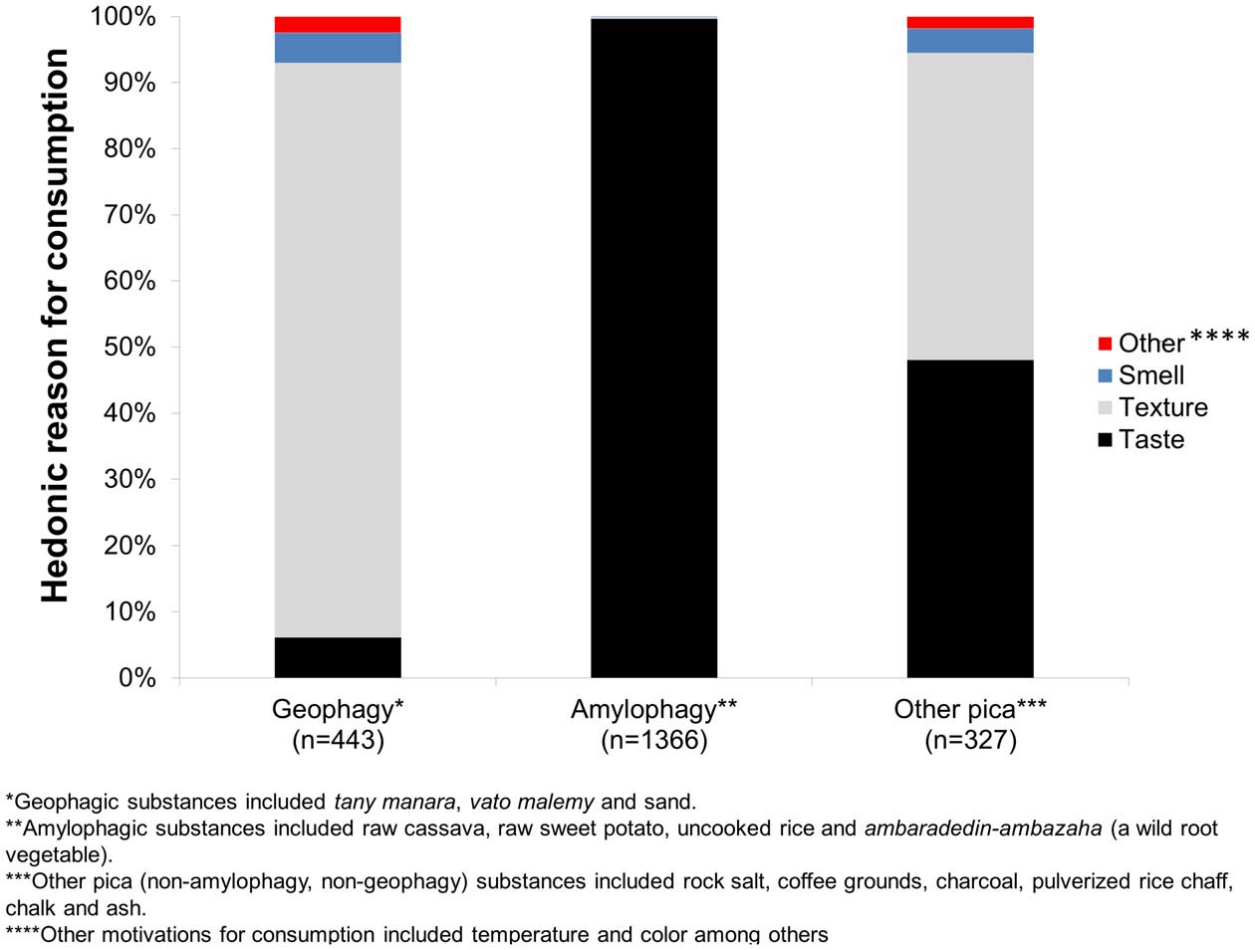
Motivations for pica behavior and amylophagy. Geophagy is largely motivated by the texture of the substances (as well as medicinal use, see Table 3) whereas amylophagy was almost entirely driven by taste. Other non-foods were jointly motivated by taste and texture. Olfactory cues played only a small role in pica behavior.

All consumers were asked to describe the hedonic appeal of the substances they ate (Fig. 3). Geophagic substances were largely consumed because of their texture (86.9%), whereas amylophagic substances were almost exclusively consumed for their taste (99.6%, Fig. 3). This parallels the local Malagasy attitude viewing raw starches as food. Other non-foods were consumed for both their taste (48.0%) and their texture (46.5%, Fig. 3). Smell played a small role in the hedonic appeal of consumption, motivating the consumption of 4.5% of geophagic substances and 3.7% of other non-foods (Fig. 3).

In addition to reasons for hedonic appeal, there was opportunity in the survey to relate information about medicinal use or other motivations for consumption. These pica substances had a wide variety of medicinal uses that were a primary incentive for consumption (Table 3). The majority of geophagy (87.3%) was attributed to medicinal use; in contrast only 0.3% of amylophagy and 21.4% of other non-foods consumption was attributed to medicinal use. The majority of geophagic behavior was attributed to spiritual, medicinal treatment and 10.3% of total pica behavior overall is attributed to the desire to obtain good luck and to protect oneself from evil (Table 3).

**Table 3:** Diversity of medicinal treatments provided through pica behavior.

Type of treatment	Percent within medicines^1^	Percent of total pica behavior^2^
Spiritual medicine that brings good luck	51.4%	10.3%
Gastro-intestinal illness	40.4%	8.0%
Other^3^	2.7%	0.6%
Cough medicine	2.5%	0.5%
Deworming medicine	1.7%	0.3%
Medicine for dehydration and fatigue	1.4%	0.3%

1 These data represent the distribution of types of medicinal treatments among those substances reported to be used for medicinal purposes. A given substance could be attributed to more than one medicinal use.

2 Total pica behavior includes geophagy and other non-foods, excluding amylophagy. These data refer to the frequencies with which respondents identified the use of a given pica substance being attributed to a given medicinal treatment.

3 The other category includes the following treatments in order of frequency: medicine for sore throats, for rotten teeth in children, against witchcraft, accidents and bad luck, for fatigue and muscle soreness, for raised and infected inflammations, enlarged testicles and/or bedwetting, anemia, healing the vagina after birth, and to induce an abortion. N.B. We have categorized diarrhea, bloody diarrhea, vomiting, bloating and gaseousness as one category called gastro-intestinal illness.

## Discussion

In this first population-based study of pica and amylophagy in Madagascar, we have demonstrated that both of these behaviors are clearly common in the Makira region, with 53.4% of men, women and children having practiced geophagy, 85.2% having practiced amylophagy and 19.0% having consumed other non-food items in the past year. These prevalences are much higher than has been reported in other population-based studies of pica and amylophagy [1]. We were confident that we received accurate and honest responses from children, adolescents and adults because of our team's long-standing relationship with the communities in the surveyed villages and our rapport with children. Also, pica is not a stigmatized behavior in this culture, a characteristic that lends itself to more accurate reporting. These may explain the higher prevalences than other settings, but it is also possible that behaviors are actually different.

This study is one of the few population-based descriptions of pica and amylophagy among men. Our results show that the behavior was widespread (male children (44.2%), adolescents (25.0%) and adults (63.0%)). As compared to the three other studies that have reported pica in men, prevalences in the Makira region were much higher with no reports of pica in men from California [32] or North Carolina [33], and a prevalence of only 11% of black South African men [34]. In contrast, in our study, prevalence varied by age but not by sex, with the exception that adolescent females (56%) seemed to practice geophagy more frequently than adolescent males (25%), although the small sample size precludes any robust conclusions to be drawn. There are a number of potential explanations for the differences in reported behavior among Malagasy adolescents and men, including the low clay content of geophagic substances. However, further studies among adolescents and adult men in other settings are necessary to know if there are actual differences or if the prevalences observed here are in fact similar.

Second, in contrast to every other study of patterns in pica of which we are aware, we found no peak in pica, amylophagy and other non-foods consumption behaviors during pregnancy. This may be because pregnancy is highly underreported by women in this region; local taboos prohibit the discussion of pregnancy before the birth of a child. With that said, the prevalence was significantly lower among pregnant women than among non-pregnant non-lactating female adults (p = 0.048) as well as among lactating women (p<0.0001). In fact, the prevalence among non-pregnant women of pica that does not include amylophagy or medicinally-motivated geophagy (25.7%) was similar to prevalences observed among *pregnant* women in sub-Saharan Africa [1]. This is striking because pica is often considered a behavior unique to pregnant women [1]. However, we cannot determine a reliable prevalence of this behavior during pregnancy because of underreporting, and thus can only reliably state that this behavior is very common amongst women.

Our results concerning amylophagy were also different than those in many other studies. Amylophagy prevalence in pregnant and lactating women has been reported across the globe, ranging from no observable amylophagic behavior to 34.6% (Illinois, USA [35]) and 36.3% (Tanzania, [22]) among pregnant women. In our study, we found a high prevalence of amylophagy in both sexes and among all ages. The prevalence was 81.5%, 80.5% and 93.4% for children, adolescents, and adults, respectively with highest rates in adult men (93.6%). The high prevalence of raw cassava consumption is of concern because of the potential toxic effects due to above-threshold concentrations of hydrogen cyanide [36].

There are further differences encountered in this study as compared to other studies of pica and amylophagy. These high prevalences may be in part attributable to the fact that local Malagasy recognize these raw starches as food and not as craved non-food substances, per se. In other words, while this is clearly amylophagy, it seems to not be pica. Similarly, a large proportion recognized the medicinal properties of non-food substances, amounting to 20% of the motivations for pica. Thus, conflating craved non-food items with those ingested for nutritional, curative, or protective purposes would lead to an overestimation of population prevalence of pica.

In terms of the physical characteristics of the samples, *tany manara*, *vato malemy* and sand were much lower in clay (2.8%, 4.1% and 20.5%, respectively) than reported in other studies of geophagy [5]. This low clay content, unusual for geophagy, may be explained by the perceived medicinal properties, arising from perceived medical efficacy rather than a craving. Finally, the olfactory properties of pica substances played little role in motivating pica behavior. Indeed, only 7.7% mentioned smell as a motivation for consumption. In many other studies, however, olfactory cues have motivated pica, especially as an impetus to eat earth [7]. Future studies should be careful to distinguish non-food consumption from pica by ascertaining the role of cravings and local definitions of “food” and medicinal use.

There are, however, some similarities to other studies of pica. The prevalence of non-food consumption in children not including amylophagy and medicinally-motivated pica (18.8%) was similar to other population-based studies among children in sub-Saharan Africa [6], [15], [16], [37]. Further, similarly high rates of geophagy have been observed among in pregnant women in South Africa [37], Ghana [31] and Nigeria [11]. Although the prevalence of geophagic behaviors may be similar, the frequency and amount consumed is lower in Madagascar than on Pemba Island, Tanzania where these data area also available [38].

There are several limitations to this work. Survey data, even when combined with ethnographic research, can only deliver a limited understanding of the true motivations of behavior. There are clear cross-sectional differences in geophagy, amylophagy and other pica behaviors in this area, as suggested by the statistically significant differences across all three types of substances and between the local Betsimisaraka and Tsimihety living in the same area. We are uncertain of the motivations underlying these differences, however. Further, although all forms of consumption were well-documented, we found it difficult to disentangle craved consumption (i.e. pica) from other types of ingestion (e.g. medicine, food). Third, pregnant women and adolescents as a whole were undersampled in the population. This is in part due to the migration of teenagers from these interior areas for schooling or employment opportunities and the hesitancy of women to discuss pregnancy until after birth. Lastly, only one sample was collected of each pica substance submitted for laboratory analysis. Future work will document the chemical properties of these substances more thoroughly.

In sum, this study is important not only because it is the first empirical evidence of pica and amylophagic behaviors in Madagascar but because it reveals new patterns in consumption. Males and non-pregnant females have rarely been included in pica studies; these findings provide incentive to document the presence of this behavior across a spectrum of ages and among both sexes. Lastly, and perhaps most importantly, with a prevalence this high, the attribution of the behavior to medicinal properties, and the potential for deleterious and protective benefits of these ingestive behaviors, a strong case has been made for further investigation of both the etiology and health consequences of earth, raw starch, and other non-food consumption

## Supporting Information

Abstract S1 (DOCX)
