# Supplementary material for: Pica and Amylophagy Are Common among Malagasy Men, Women and Children
Source: PLoS One. 2012 Oct 17;7(10):e47129. doi: 10.1371/journal.pone.0047129 (PMC3474809; doi:10.1371/journal.pone.0047129)
Supplement: Abstract S1 — (DOCX) [file pone.0047129.s001.docx]

Pica, la consommation soif et téléologique de substances non alimentaires, est une préoccupation de santé publique pour ses éventuelles conséquences néfastes sur la santé et de salubrité. Cependant, ni sa prévalence, ni les corrélats sociaux et biologiques n’ont été bien caractérisés. Par conséquent, nous avons mené la première étude de population de pica à Madagascar. De Février à Décembre 2009, nous avons sondé les comportements pica dans un échantillon aléatoire de 760 individus plus âgé que 5 ans dans 167 ménages parmi les deux groupes ethniques dans 16 villages de la zone protégée de Makira de Madagascar. Parmi les 760 personnes interrogées, 62,5% étaient enfants (5-11 ans), 5,4% étaient adolescents (12-16 ans), et 35,1% étaient adultes (> 17 ans). Treize articles non alimentaires ont été signalés d'être consommée. Ensemble de la population de l'année précédente, la prévalence de la géophagie était de 53,4%, de amylophagy, 85,2%, et de substances pica autres (par exemple du charbon de bois, de la craie) était de 19,0%. La prévalence de ces comportements n'est pas plus élevée pendant la grossesse. Cette étude diffère des études précédentes en termes de prévalence plus élevée d'ensemble de ces comportements, la prévalence élevée chez les hommes, et l'absence de tout pic dans les comportements pendant la grossesse. Toutefois, le pica est caractérisé par un besoin et il y a deux catégories de substances qui élèvent nos estimations, mais n'entrent pas dans la définition stricte: 1) les substances consommées pour l'auto-médication et 2) les substances considérées comme des aliments, tels que toutes les substances amylophagic dans ce cas. Nos résultats suggèrent que les études de population de pica devraient inclure les hommes de tous âges. En outre, la prévalence du comportement souligne l'importance de la compréhension de l'étiologie et les conséquences sanitaires de ces comportements ingestifs.
